# Monitoring military pilots with textile sensors: physiological responses and signal quality under extreme conditions

**DOI:** 10.3389/fphys.2026.1911780

**Published:** 2026-09-14

**Authors:** Aline F. Renz, René M. Rossi, Frederik Bauer, Andreas Stier, Denis Bron, Simon Annaheim

**Affiliations:** 1 Laboratory for Biomimetic Membranes and Textiles, Empa, Swiss Laboratories for Materials Science and Technology, St. Gallen, Switzerland; 2 Department of Health Sciences and Technology, Swiss Federal Institute of Technology (ETH), Zurich, Switzerland; 3 Armasuisse, Bern, Switzerland; 4 Aeromedical Center, Swiss Air Force, Dubendorf, Switzerland

**Keywords:** aerospace, breathing, ECG, extreme conditions, fighter jet pilot, military, signal quality

## Abstract

**Introduction:**

Military pilots are exposed to severe physiological challenges during their missions that may affect cognitive and physical performance. Therefore, continuous monitoring potentially provides critical insights about the impact of extreme condition exposure on the fitness to fly of military pilots.

**Methods:**

We applied a textile-based monitoring system during training sessions with pilot aspirants to investigate the impact of hypobaric hypoxia and high G-force exposures on the signal quality obtained for a 1-lead electrocardiogram (ECG) and chest excursions. The physiological variables considered were heart rate, heart rate variability, as well as respiratory frequency and respiratory amplitude.

**Results:**

In general, 93.4% and 90.4% of the recorded ECG time segments during hypoxia and G-force exposure, respectively, were classified as plausible for further analysis. For respiratory data, 70.7% and 81.3% were classified as accurate for further data analysis and interpretation.

**Discussion:**

Detailed information about signal quality was found to be critical for assessing physiological variables recorded in extreme conditions, including distinguishing between intra- and interpersonal variability. Furthermore, the combination of various physiological signals allows for a more holistic interpretation of body responses, for the assessment of body tolerances and an early detection of possible physical and cognitive impairments.

## 1 Introduction

Military pilots are exposed to severe physiological challenges that may have an impact on their physical and cognitive performance (Aircraft Technology, 2018). Exposures such as high multiaxial g-forces and rotation, hypoxic conditions, radiation sources and high temperatures, or vibration and noise can lead to a reduction in their physical and mental performance, as demonstrated for severe hypoxic exposures (Romer et al., 2007).

This extraordinary physical strain requires increased physical and mental efforts of the pilot to cope with stressors and to perform the flight tasks at a high level of accuracy. A higher activation level of pilots results in increased respiratory activity and an increase in heart rate (Truszczyński et al., 2009), and a decrease in heart rate variability (HRV) (Vigo et al., 2010). Furthermore, blood lactate concentration during hyperpnea was found to be higher in hypoxia (Verges et al., 2010). Bustamante-Sánchez et al. (Bustamante-Sánchez et al., 2021) analyzed the psychophysiological responses of the participants in incremental hypoxia training (3 minutes at sea level, 8 minutes at 5,000 meters, and a maximum of 10 minutes at 7500 meters above sea level. They reported statistically significant decreases in blood oxygen saturation and increases in heart rate mean values during hypoxia. Also, a significant increase in the rating of perceived exertion was reported during hypoxia. Impairments of cognitive performance have also frequently been reported under high-altitude exposures (McMorris et al., 2017).

G-force induced loss of consciousness (G-LOC) is caused by G acceleration stress and is one of the most life-threatening problems for pilots (Choi et al). A study from the UK Royal Air Force in 2017 revealed that 120 (14.8%) aircrew reported at least one episode of G-LOC, and in 260 cases (32.2%), at least one episode of almost loss of consciousness (A-LOC) occurred (Slungaard et al., 2017). The authors showed that the prevalence of reported G-LOC had decreased compared to an earlier survey in 2005 and concluded that this result might be due to the introduction of centrifuge training. The tolerance of the human body to acceleration forces depends on their magnitude, direction and duration, as well as individual factors like age or body weight (Aircraft Technology, 2018).

To prevent such events, early warning algorithms based on electromyogram (EMG) measurements have been proposed (Kim et al., 2017). However, analyses using EMG sensors were restricted to training situations only. Other warning signs proposed are, for example, non-contact measurement systems detecting changes in eye blink rate and head position (Kutilek et al., 2019). A study employing multi-sensory data collection during flight simulations has established a more nuanced understanding of how pilots respond to sudden, critical exposures. The study analyzed subjective feedback from pilots and objective physiological data, creating a reliable index for distress levels and performance capabilities during critical flight phases (Scannella et al., 2018; Xing et al., 2023). This method substantiates the need for a multidisciplinary approach to pilot evaluation, blending human factors with technology to enhance flight safety. However, the integration of well-validated bio-monitoring systems capable of recording changes in vital signs like heart rate, body temperature, respiration and body motion has not been considered so far (Kim and Koh, 2025). The measurement of physiological parameters, e.g. for the assessment of cognitive task load, has been proposed in several studies since the 1960’s, but the identification of reliable predictors for a reduction in mental or physical performance has been limited by the use of conventional physiological monitoring technologies (Yang et al., 2024). Recent studies investigated the possibility of using wearable sensors to assess vital signs like heart rate, respiratory rate or oxygen saturation (Coffey et al., 2024). Such devices have been commonly used for several years in sports or recreational activities, but not for medical diagnostics due to higher demands for the accuracy and reliability of the systems. The use of multimodal data to predict soldiers’ health and readiness was repeatedly proposed by (De Vries et al., 2025) while highlighting the need for an accurate assessment based on high-quality data.

Challenges of using wearable sensors to assess vital signs in aviation include sensor accuracy, robustness to reliably record vital signs in extreme conditions, integration into flight suits, and interference with the pilot’s equipment (Shaw et al., 2024). Therefore, continuous evaluation of the signal quality from sensor recordings is crucial for interpreting and assessing the pilot’s readiness. However, signal quality assessments specifically under extreme or operationally demanding environmental conditions are rarely addressed in recent literature (Lecture Notes in Civil Engineering, 2021; Chen et al., 2022). Data on the impact of extreme environmental working conditions on system output, and on the accuracy of subsequent physiological assessments derived from it, therefore remain limited. For field applications, the reliability and robustness of the system are as critical aspects for the acceptance of new wearable technologies (De Vries et al., 2025), as their ease of use and predicted usefulness (Kelly and Gilbert, 2018). The relevance of monitoring vital signs and the validity of the predictive models used as early warning parameters has yet to be shown.

Among the different types of wearable health monitoring systems, textile-based sensors have emerged as an interesting technology due to their flexibility, softness, breathability, compatibility with flight suits, and conformation to each body location, which increases users’ comfort and acceptance, allowing continuous monitoring for extended durations (Li et al., 2023). However, as these systems are usually not directly attached to the skin, their reliability may suffer from motion artefacts (An and Stylios, 2020). Consequently, validation is required for each foreseen use case. Our group has very recently validated a textile-based electrocardiogram (ECG) and respiratory monitoring system for sleep apnea monitoring (Baty et al., 2024). The goal of the present study was to assess the reliability and accuracy of a similar monitoring system for potential integration into a medical early warning system designed to monitor pilots and soldiers. This system was worn by the pilots during low-pressure and high-G exposure training.

## 2 Materials and methods

In the current study, the accuracy and relevance of employing a textile-based monitoring system for monitoring pilots under extreme conditions were evaluated. The monitoring activities were conducted during low-pressure exposure in a chamber and high-G exposure in a high-performance three-axis centrifuge. For the study, a total of 10 healthy pilot aspirants with an average age of 22.6 years (standard deviation [SD]: 2.2y) volunteered to wear the monitoring system during their regular training and qualification blocks, which were conducted according to standard protocols of the Swiss Aeromedical Center. The monitoring of the pilots’ vital parameters (ECG, respiration) were conducted in accordance with the Declaration of Helsinki (World Medical Association, 2025). The Institutional Review Board of the Aeromedical Center of the Swiss Air Force approved the conduct of routine training and qualification modules that are part of pilot education, including the concomitant monitoring of vital signs, and the participants provided written informed consent.

### 2.1 Textile monitoring system

Throughout the experimental exposures, participants were equipped with a textile monitoring system (**Supplementary Figure 1**) comprising three distinct sensor types integrated into a belt worn around the chest. The system included two knitted ECG electrodes for 1-lead ECG recording for the calculation of heart rate (HR) and heart rate variability (HRV). In addition, a conductive stretch-sensitive textile sensor was applied to measure changes in chest excursion. Based on these measurements, respiratory frequency (f_resp_) and the respiratory depth (referred to as respiratory sensor elongation amplitude [amp_resp_]) were determined. The third sensor type measured skin heat flux in the chest region and was not considered for further analysis in the current study. The 1-lead ECG system was previously clinically validated with 242 patients and compared to gel electrodes (Fontana et al., 2019). A similar combined system of ECG and respiratory monitoring was also used and validated for apnea monitoring and diagnostics (Baty et al., 2024). The data was recorded using a custom-made data logger placed in a pocket at the lower abdomen. Respiration was recorded at 25 Hz (Texas Instruments ADS131M08), while the ECG was recorded at 512 Hz (NeuroSky BMD101). Additionally, the logger was equipped with an Inertial Measurement Unit (IMU; Analog Devices ADXL345) to measure the acceleration in all spatial directions. To identify disturbances in signal quality motion artefacts, a recently developed method for signal quality assessment was applied in the present study (Martins et al., 2025). The method considers the entire PQRST complex of the ECG signal. The individual QRS and PQRST complexes are averaged over 15s-time segments. The similarity between each complex and the average value of the complexes (considered as the reference complex for a specific time segment) is calculated using the Pearson correlation analysis. As a third parameter, the standard deviation of the RR-intervals in the measured segments is determined, and the number of outliers is assessed. We defined the plausibility of RR-intervals in a time segment to be true when the standard deviation of RR-intervals was between 1 and 200ms and no more than 2 outliers were detected (i.e. values corresponding to heart rates over 180 or under 30 beats per minute). This allowed classification of signal quality into 4 classes, based on the mean values of all Pearson correlation coefficients (PCCs) within the ECG time segment and plausibility criteria (**Table 1**). Classes 1 and 2 were considered suitable for high-precision RR-interval analysis and were therefore included in HRV analyses, whereas classes 1–3 were deemed acceptable for HR analysis. In the current study, SDNN was computed and evaluated as the only HRV parameter. As an initial quality control step, participants were excluded from further analysis if more than 50% of their data across all selected time segments were classified as outliers, ensuring a high overall data quality.

**Table 1:** Definition of the signal quality classes using the Pearson correlation coefficients PCC) for the QRS and PQRST complexes of the ECG signal and the plausibility criteria for the RR intervals (Martins et al., 2025).

Class	PCC for QRS complexes	PCC for PQRST complexes	Plausibility check
1	*≥* 0.98	*≥* 0.98	TRUE
2	*≥* 0.98	*<* 0.98	TRUE
3	*<* 0.98	*<* 0.98	TRUE
4	any	any	FALSE

Similarly, the median value of single chest excursion cycles over 30-second time segments was calculated to assess the signal quality of respiration. The similarity of chest excursion cycles with the reference median value for a particular time segment was determined based on PCC. The signal was classified into three quality classes based on mean PCC values across respiratory time segments: Class 1: PCC ≥ 0.85; Class 2: 0.75 < PCC < 0.85; and Class 3: PCC ≤ 0.75. Class 1 and 2 were considered suitable for further analysis. Subjects were excluded from further analysis if more than 50% of the time segments were classified as Class 3.

### 2.2 Hypobaric hypoxia exposure

The military pilot trainee underwent a standard training commonly practiced at the Aeromedical Center of the Swiss Air Force (FAI Dübendorf, Switzerland) in a low-pressure chamber, simulating an aviation scenario involving a loss of cabin pressure. The participants were grouped in pairs of two for the exposure sessions, with a medical doctor closely supervising each session. Before the exposure, they underwent preoxygenation using aviation oxygen masks (AOM). Throughout the experiment, the AOM was intermittently removed for specific periods of time according to the protocol (**Table 2**; **Figure 1**). During AOM-off periods, the participants were instructed to perform simple calculation tasks and respond to questions from the medical doctor to assess their cognitive performance. The numerical analysis of the data from the wearable system encompassed nine distinct data intervals, each with a duration of 30 seconds, denoted i1 to i9 (**Figure 2**). The 30-second time windows were selected for HRV analysis based on previously reported suitability of 30-second time intervals for ultrashort HRV insight (Pecchia et al., 2018; Shaffer et al., 2020). Data interval 1 (i1) captured the timeframe at the end of Phase 2 (ascend with AOM applied, **Table 2**). Phase 3 consists of the continuing ascent to 7500m asl and staying at this altitude without AOM. Data interval 2 (i2) includes data at the beginning of phase 3, while data interval 3 (i3) and 4 (i4) consist of data just before and after the AOM was reapplied (indicating the end of phase 3). Data interval 5 (i5) and 6 (i6) consider the time before and after the second removal of the AOM at an altitude of 9000m asl, and data interval 7 (i7) and 8 (i8) account for the 30 seconds just before and after the AOM was reapplied again at the end of Phase 5. Data interval 9 (i9) focuses on the time 60 seconds after Phase 5 concluded. Phases 3 and 5 consisted of hypoxic conditions.

**Table 2:** Hypobaric hypoxia exposition protocol.

Phases	Description
Phase 1	Rise to 2500m above sea level (asl) and back to local altitude (440m asl) with aviation oxygen mask (AOM) on for test of equipment and pressure equalization.
Phase 2	Rise to 4500m asl at 5.55m/s ascent with AOM on.
Phase 3	At an altitude of 4500m asl, the AOM was removed and the participant was instructed to start the calculation exercise. The altitude was gradually increased to 7500m asl, during which the AOM remained off until the medical doctor intervened and put the AOM back on.
Phase 4	Rise to 9000m asl at 5.55m/s ascent with AOM on.
Phase 5	At the altitude of 9000m, the AOM were removed again, and the participants continued the calculation exercise until the medical doctor put the AOM back on.
Phase 6	Decline back to local altitude with AOM on.

The altitude information refers to the altitude that corresponds to the air pressure in the chamber.

**Figure 1:**
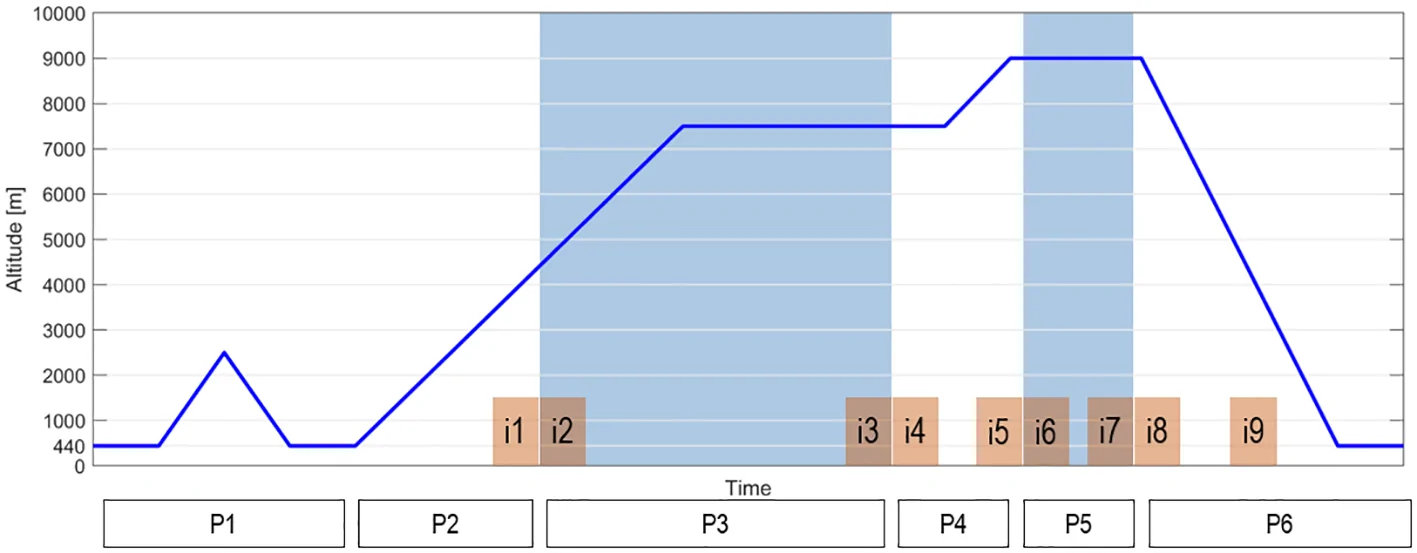
Simulated altitude profile. The blue periods highlight the phases when the participants had their aviator oxygen masks removed (phases 3 and 5). Details regarding the phases are available in **Table 2**. The orange annotations (i1 to i6) indicate the specific 30-second measurement intervals for ECG and breathing signals.

**Figure 2:**
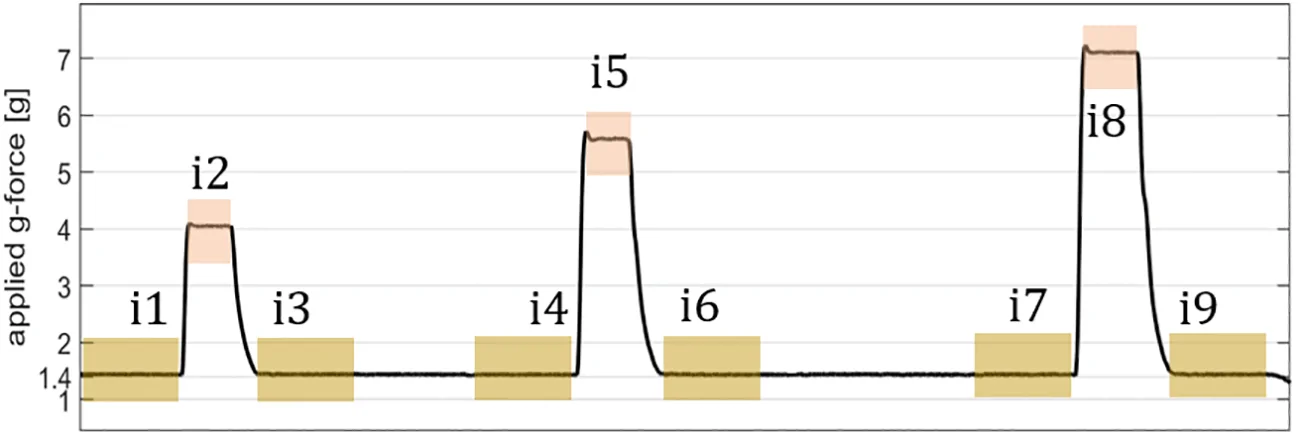
G-force exposure protocol for the qualification profile. The intervals i1 to i9 were used for the numeric analysis of the wearable system data (ECG and breathing signals). The yellow annotations (i1 to i9) indicate the specific 30-second measurement intervals for ECG and breathing signals with exception for i2, i5 and i8 in orange representing the 15-second high-G exposure intervals.

### 2.3 High-G exposures

The participants underwent high-G exposures in a high-performance three-axis centrifuge at the Center for Aerospace Medicine in Königsbrück, Germany, to receive G-force qualification for military jet pilots. For this, military pilot trainees had to successfully complete a 7G exposure for 15-second duration (according to NATO STANAG 3827). The 7G exposure was preceded by two exposures of 4G and 5.5G separated by baseline exposures of 1.4G with individual durations between 1 and 2 minutes (**Figure 2**). The numerical analysis of the data from the wearable system encompassed nine distinct data intervals (i1 – Fi9) with an identical duration of 15 seconds during the high-G exposure (i2, i5, i8) and respective 30 second intervals just before and right after high-G exposures. As for the hypobaric hypoxia exposure 30 second time windows allow for ultra short HRV insight.

### 2.4 Statistical analysis

A univariate repeated-measures ANOVA with *post hoc* tests corrected using Tukey’s method was conducted for ECG (HR, SDNN) and respiration-related parameters (f_resp_, amp_resp_). Data sets were checked for sphericity, homogeneity and normality. Corrections were applied to data sets that did not meet sphericity requirements (Greenhouse-Geisser; corrected data sets are indicated in the text). The Jamovi software was used for statistical analysis (The jamovi project, 2024). The level of significance was set at 0.05.

## 3 Results

### 3.1 Hypobaric hypoxia exposures

The quality of the ECG signals obtained during the hypobaric hypoxia exposure of the participants was assessed and classified for each interval (**Table 1**). A total of 93.4% of the selected ECG time segments were assigned to classes 1-3, indicating good suitability for further HR analysis across all participants (**Table 3**). For the HRV analysis, 2 participants had to be excluded in line with our 50% threshold (**Supplementary Figure 2**). With this exclusion, as seen in **Supplementary Table 1**, only 2.6% of the data remained below this threshold. The assessment of the chest excursion extracted respiratory signals identified 70.7% of respiratory time segments rated as class 1 or 2 after exclusion of 5 participants from further evaluation due to insufficient data quality (**Supplementary Figure 3**).

**Table 3:** The mean ECG (ECG SQ) and respiratory signal quality (Resp SQ) and associated standard deviation (SD) for time segments assigned to classes 1 to 4 (ECG SQ, n=10) and classes 1 to 3 (Resp SQ, n=5) during hypobaric hypoxia exposure (see section 2.1 and **Table 1** and for detailed assignment criteria).

ECG SQ	Mean	SD	Resp SQ	Mean	SD
Class 1	58.8%	43.5%	Class 1	32.8%	41.6%
Class 2	26.4%	33.5%	Class 2	37.9%	41.8%
Class 3	8.2%	22.3%	Class 3	29.2%	39.3%
Class 4	6.6%	21.1%			

Statistically significant changes were detected in HR (Greenhouse-Geisser corrected p<0.001) and SDNN (Greenhouse-Geisser corrected p=0.005) as highlighted in **Figure 3**. No change in HR (+2%, p=1.0) was observed right after the initial removal of the AOM (i2). SDNN dropped by 27% but not statistically significant (p=0.26). At the end of the first hypoxia exposure at 7500m asl (i3), both HR and SDNN showed significant changes compared to i1: +33% (p<0.001) and -63% (p=0.019), respectively. The second hypoxia exposure at 9000m asl resulted in a shorter exposure time (i6-i7), with no change in HR (0.66%, p=1.0) and a drop in SDNN by -23% (p=0.045) upon removal of the AOM (i6). A significant drop could also be monitored for SDNN when comparing pre-AOM removal interval i5 to i7 (-53%, p=0.011) and again upon AOM application (SDNN 94%, p=0.02) (i8, **Figure 3C**). For HR the changes were mainly noticeable for i5-i7 (19.8%, p=0.054), whereas i7-i8 remained stable (-2%, p=0.75).

**Figure 3:**
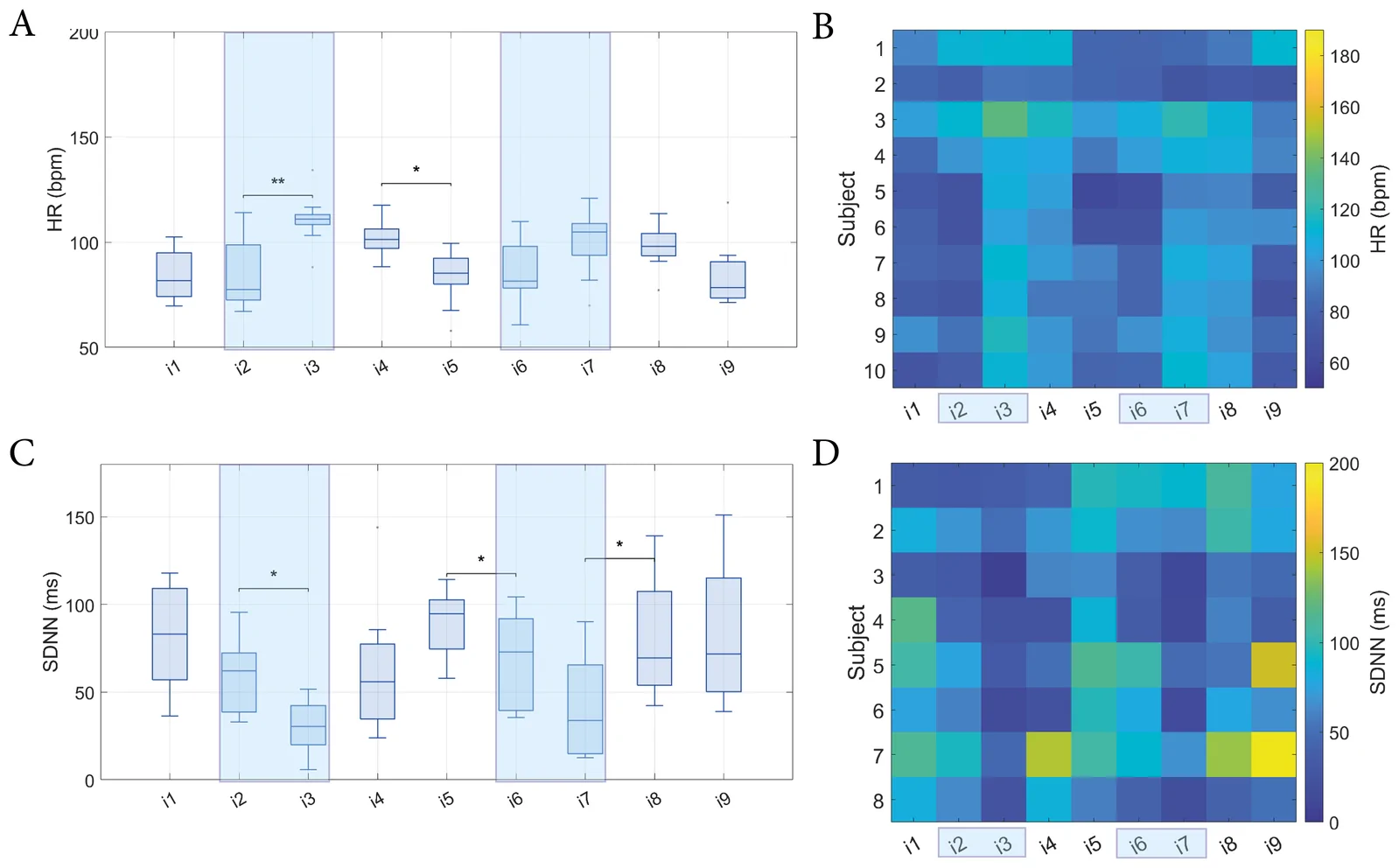
Heart rate and heart rate variability for various measurement intervals (i1 – i9; further information on the prevailing conditions during the individual measurement intervals can be found in **Figure 1**) during hypobaric hypoxia exposure (*p ≤ 0.05, **p≤ 0.01). **(A)** Median HR across n = 10 participants for each interval (Hypoxic conditions highlighted in blue), with marked significance for consecutive changes in mean HR. **(B)** Overview of participant-based mean HR for each interval. **(C)** Median SDNN across n=8 for each interval (Hypoxic conditions highlighted in blue), with marked significance for consecutive changes in mean SDNN. **(D)** Overview of participant-based mean SDNN for each interval.

Due to the low subject number and resulting degrees of freedom for the respiratory data no statistical evaluation was conducted for respiratory frequency and respiratory sensor elongation amplitude (**Figure 4A**). Nevertheless, remarkable changes were observed at the end of the first hypoxia exposure at 7500m asl (i3) for f_resp_ (+23.8%) while amp_resp_ stayed more constant (-5.5%). On the other hand, upon re-application of the AOM (i4) the f_resp_ value returned immediately to pre-exposure levels (-0.2%), while amp_resp_ increased significantly by 51.3%, when compared to i1. The second hypoxia exposure at 9000m asl (i7) revealed more distinct changes in both f_resp_ (+40.7%) and amp_resp_ (-19.2%). Upon the re-application of the AOM (i8), values returned more slowly to pre-exposure values for f_resp_ (+24.6%) and showed a steep increase for amp_resp_ (+40%). This trend was observed across most intra-individual data, while for respiratory amplitude two distinct patterns emerged (**Figure 4B**).

**Figure 4:**
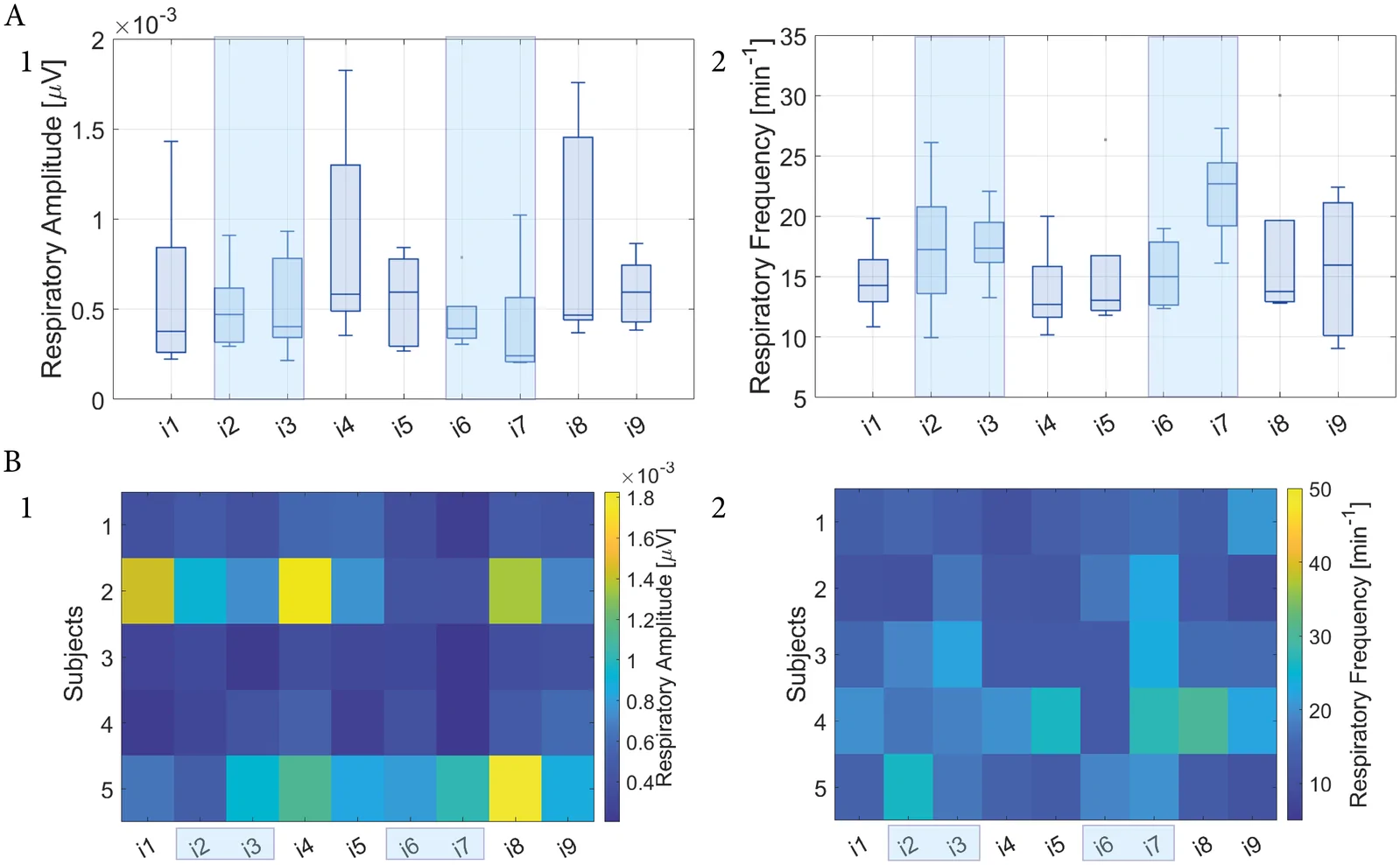
Respiratory frequency and respiratory sensor elongation amplitude for various measurement intervals (i1 – i9; further information on the prevailing conditions during the individual measurement intervals can be found in **Figure 1**) during hypobaric hypoxia exposure (Hypoxic conditions highlighted in blue). **(A1)** Shows the median for the respiratory amplitude across all intervals for n=6. **(A2)** Shows the median for the respiratory frequency across all intervals for n=6. **(B1)** Heatmap for interpersonal variability of respiratory amplitude for each interval (n=6). **(B2)** Heatmap for interpersonal variability of respiratory frequency for each interval (n=6).

### 3.2 High G-exposures

A total of 90.4% of the selected ECG time segments and 81.3% of the selected respiratory time segments were eligible for further analysis (**Table 4**), where 2 participants had to be excluded for the HRV analysis. In addition, the actual high G-exposure intervals i2, i5, and i8 were not suitable for HRV analysis due to the shorter time segments and the lack of Class 1 and 2 classification (**Supplementary Table 2**). For the respiratory analysis, 1 participant had to be excluded in line with our threshold (**Supplementary Figure 5**).

**Table 4:** The mean ECG (ECG SQ) and respiratory signal quality (Resp SQ) and associated standard deviation (SD) for time segments assigned to classes 1 to 4 (ECG SQ, n=9) and classes 1 to 3 (Resp SQ, n=9) observed in participants during high-G exposition (see section 2.1 and **Table 1** and for detailed assignment criteria).

ECG SQ	Mean	SD	Resp SQ	Mean	SD
Class 1	14.3%	23.1%	Class 1	54.3%	43.3%
Class 2	71%	40.8%	Class 2	27%	39.7%
Class 3	5.1%	30%	Class 3	18.7%	34.6%
Class 4	9.6%	37.6%			

A significant change in HR was found during repeated exposures to high G-forces (Greenhouse-Geisser corrected p<0.001), while no effect was observed for SDNN (p=0.5). The changes in HR from pre-to-post high G-exposure were significant for each exposure interval, with confidence ranging from p<0.001 to p=0.017 (**Figure 5A**). When looking into the intra-subject variability for each interval (**Figure 5B**), this behavior is reflected, and a clear increase from pre to high G-exposure is indicated. When looking into post-exposure responses, HR was significantly increased when compared to i1, with 17.6% p=0.002, 26.8% p<0.001, 32% p<0.001 after 4G. 5.5G and 7G exposures, respectively. (**Figure 5C**). For SDNN, a high variation was observed, particularly during and right after high G-exposure (**Figure 5D**). Particularly during high G-exposure intervals, a reduction in ECG signal quality was observed (**Supplementary Figure 4**).

**Figure 5:**
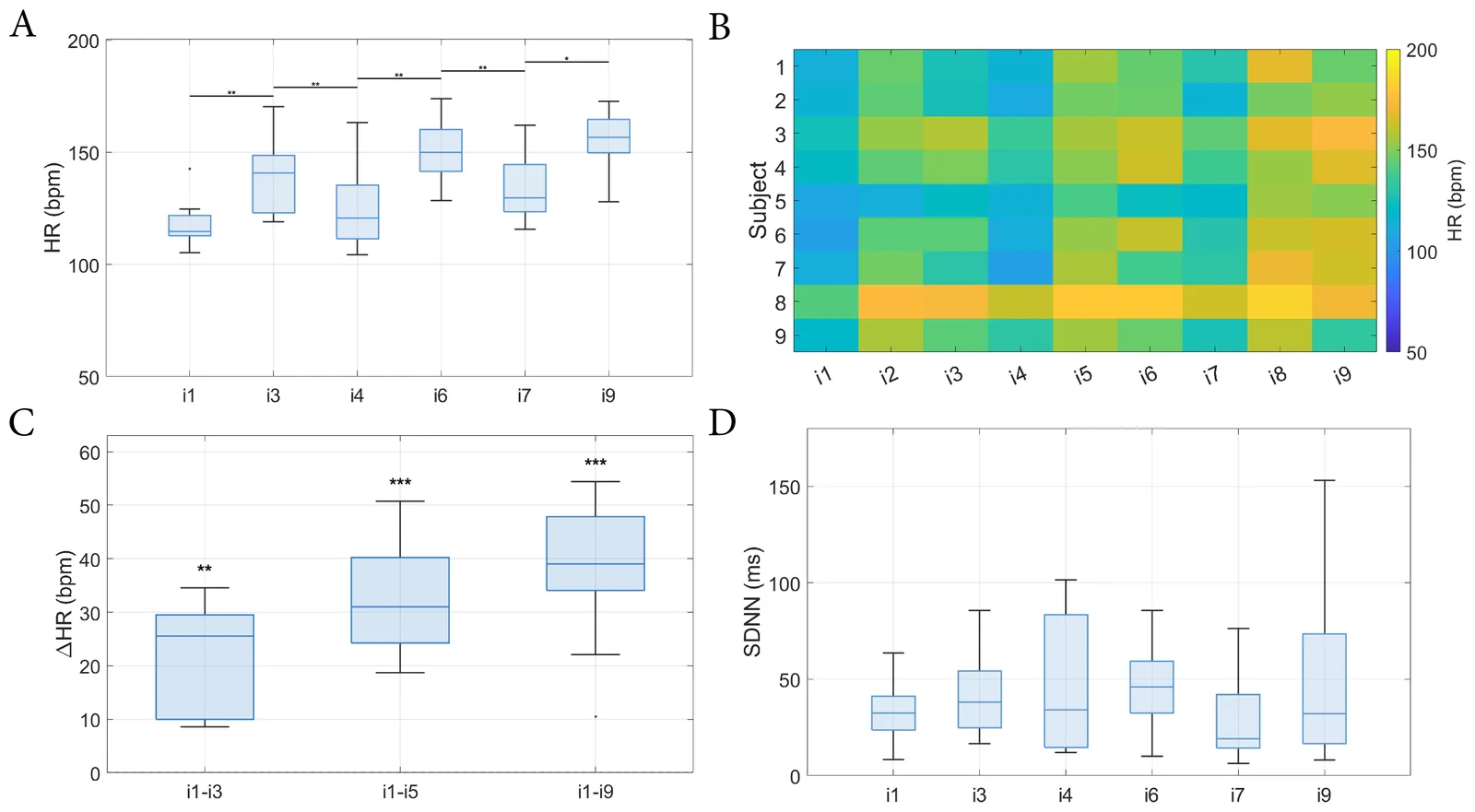
Heart rate and heart rate variability for various measurement intervals (i1 – i9; further information on the prevailing conditions during the individual measurement intervals can be found in section 2.3, **Figure 2**) during high G-exposures (*p ≤ 0.05, **p≤ 0.01, ***p ≤ 0.001). **(A)** Median HR across n=9 for pre and post high G-exposure intervals, with marked significance for consecutive changes in mean HR. **(B)** Overview of participant-based mean HR for each interval. **(C)** Overview of changes in HR from i1 to post-G-Exposure, with mean significance level. **(D)** Mean SDNN across n=8 participants for pre and post high G-exposure intervals.

Even though a statistically significant effect was detected for amp_resp_ (p=0.014), no significant differences were identified between subsequent data intervals (**Figure 6A1**). A clear trend was observed in the intra-subject investigation (**Figure 6B1**) with decreasing amplitude during each high G-exposure event when compared to i1 (-23.4% for i2, -47.7% for i5, -65.6% for i8). For f_resp_ no statistically significant effect was found. A decreasing f_resp_ (-20.7% for i2, -25.8% for i5, -31.3% for i8) was observed when compared to i1. The changes in amp_resp_ were more distinct and aligned more closely with intra-subject changes when compared to f_resp_ values, where a distinct change during the 5.5 and 7 G-exposure was observed for only 3 participants (**Figure 6B**).

**Figure 6:**
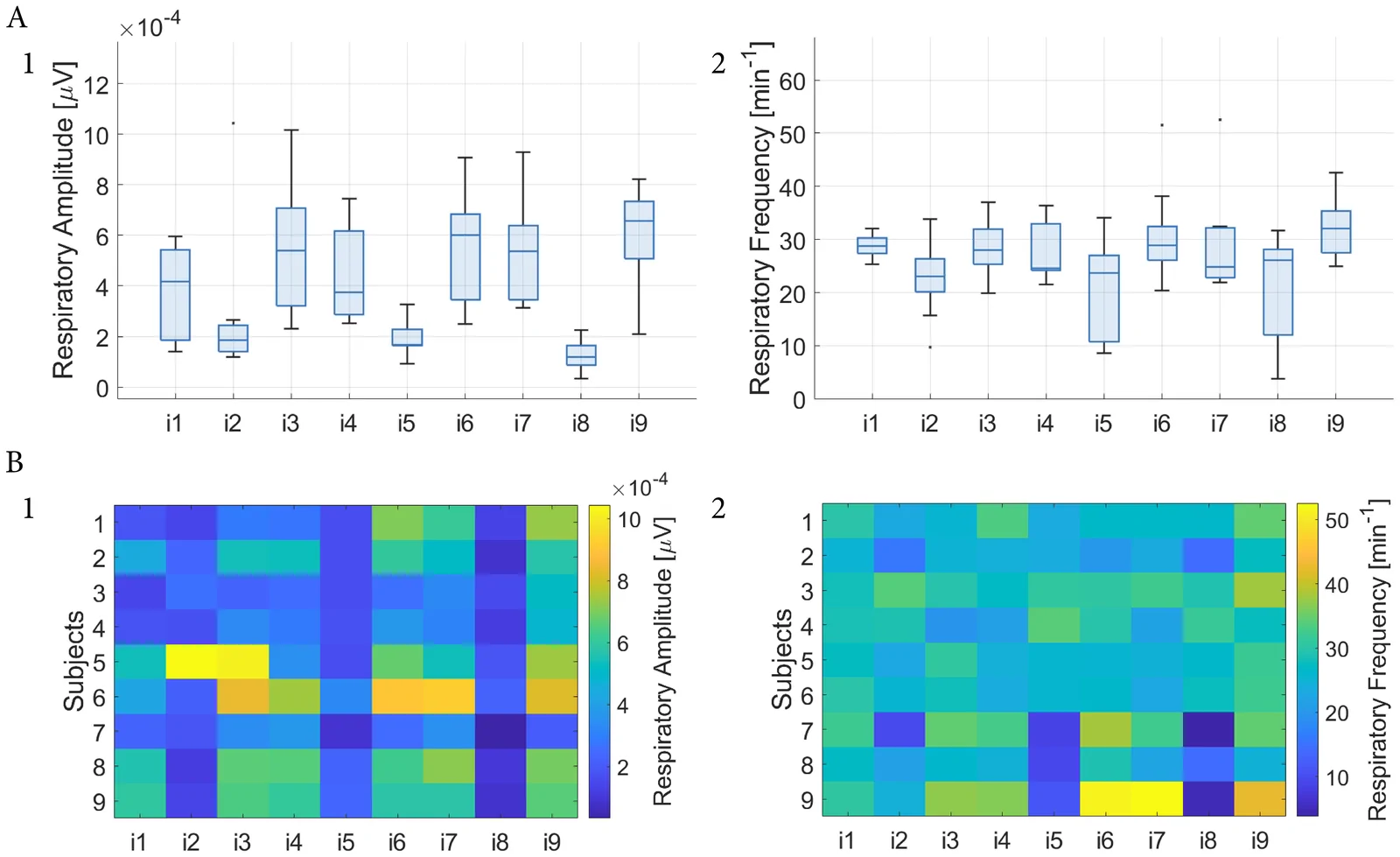
Respiratory frequency and respiratory sensor elongation amplitude for various measurement intervals (i1 – i9; further information on the prevailing conditions during the individual measurement intervals can be found in **Figure 2**) during high G-exposures. **(A1)** Shows the median for the respiratory amplitude across all intervals for n=9. **(A2)** Shows the median for the respiratory frequency across all intervals for n=9. **(B1)** Heatmap for interpersonal variability of respiratory amplitude for each interval (n=9). **(B2)** Heatmap for interpersonal variability of respiratory frequency for each interval (n=9).

## 4 Discussion

The textile monitoring system investigated achieved 93.4% and 90.4% of selected ECG time segments meeting the plausibility criteria (signal quality classes 1–3) during hypobaric hypoxia (n=10) and high G-force (n=9) exposure, respectively. In the hypobaric chamber, 85.2% of ECG signals across all participants and intervals were classified as class 1 or 2, while it’s 85.3% in the centrifuge with n = 9. We assume that the ECG signal was affected by an increased muscular activity (mainly legs and torso) during high G-exposure, and the Valsalva maneuvers resulted in increased muscular activity in the torso. This affected the electrophysiological recording of the ECG signal (i.e., superimposition of the ECG signal on increased electromyographic activity) and could explain the apparent contradiction of detecting no significant changes in SDNN across the intervals. Furthermore, the exposure to high accelerations in the centrifuge might also lead to motion artifacts. As a result, a reduction in signal quality was particularly observed during high G-exposure phases. Neglecting the high G-Exposure intervals and excluding the two participants not reaching the SQA threshold for HRV, the ECG signal in classes 1 and 2 rose to 97.4% and 88.8%, for the hypobaric chamber and centrifuge, respectively (**Supplementary Tables 1**, **2**). Military pilot trainees were seated in the low-pressure chamber with no acceleration. A calm body posture produced less noise, resulting in higher data quality throughout the ECG recordings. Consequently, only 6.6% of the ECG time segments were classified in class 4 during hypoxia exposure and had to be discarded for further analysis. For the HRV analysis, where only classes 1 and 2 were suitable, 2 participants had to be excluded, as highlighted in **Supplementary Figure 2**, reducing the total class 4 segments to 2.6% for the included data sets. With additional muscular activity, movement, and force-related implications during high G-Exposure, with up to 24% of signals in class 4. The intervals i2, i5 and i8 of the ECG recordings had to be discarded for further analysis. When focusing on pre- and post-exposure, neglecting one participant, the class 4 allocation dropped to 9.6%. Subsequently, for HRV analysis of high G-exposure values, an additional participant had to be excluded due to low class 1 and 2 allocations, reducing the respective class 4 allocation for HRV analysis down to 5.4%.

For respiration, 70.7% and 77.8% of plausible respiratory time segments were identified during hypobaric hypoxia (n=6) and high G-force (n=9) exposure, respectively. 4 and 1 participant had to be excluded from the analysis, respectively, as they did not meet the threshold criteria (**Supplementary Figures 3**, **5**). The respiratory signals recorded with the same textile monitoring system did differ between the two exposure modalities for class 1 (32.8 ± 41.6% during hypoxia exposure and 54.3 ± 43.3% during high G-exposure) and class 2 (37.9 ± 41.8% and 27 ± 39.7% for hypoxia and high-G exposure, respectively). This was somewhat surprising, as slightly reduced signal quality was expected during high-G exposures due to movement artifacts and changes in respiratory patterns, particularly a reduction in respiratory amplitude (**Figure 6**). These findings prove that the respiratory sensor measured chest excursions reliably, irrespective of increased muscular activity in the torso or motion artifacts during high G-exposure. However, the limited signal quality for respiratory signals during hypobaric hypoxia exposure is most likely attributed to two factors. First, overall movement artifact, as the pilots are not restricted while experiencing increased stress due to air limitations and the requirement for continuous writing. Second, the friction between the shirt and the chest excursion sensor highlighted by the challenge of the signal quality algorithm for peak detection during inhaling but not exhaling.

Nevertheless, a high inter-participant variability was observed in data acquisition accuracy, resulting in standard deviations reaching 20%-40% for the ECG signal quality in both exposure modalities and around 30%-40% for the respiratory signal quality. The high variability is, on the one hand, impacted by the overall small sample size and on the other by the relatively small-time windows each interval represents. As this won’t fully explain variability, we assume that individual factors, such as the fit of the monitoring system and skin properties, affect the measurement quality and need to be considered in more depth in future studies.

During the two high-altitude phases simulating 7500 and 9000 m asl without AOM, heart rate increased (**Figure 3A**), accompanied by a significant decrease in SDNN (**Figure 3C**), which is consistent with findings from previous studies (Bustamante-Sánchez et al., 2021). This was also detectable in the intrapersonal analysis (**Figures 3B, D**). For the respiratory patterns, the highest respiratory frequencies were found at the end of the hypoxia phases (i3 and i7); however, these changes did not reach statistical significance. Respiration immediately normalized after the application of the AOM, indicated by a reduction in respiratory frequency and an increase in respiratory amplitude. At the same time, an increase in SDNN was also observed during interval 9, indicating that military pilot trainees recovered very quickly from hypoxic stress with oxygen administration. As heart rate variability was immediately affected by hypoxic exposure and heart rate revealed significant changes, it may be concluded that these parameters can be used to monitor the physiological stress induced by hypoxia, while respiratory patterns are not clear indicators and need more data to determine its full potential.

During the high-G exposures, significant differences in heart rate were found. The changes in respiratory patterns were expected due to the breathing maneuvers to maintain cerebral blood flow, which clearly reduced the respiratory amplitude. The increase in HR could be expected as well, as this was already demonstrated in several studies (e.g (Metin et al., 2023). The increase in HR seems to be related to the level of acceleration, as the mean value reached 147 ± 16 bpm, 155 ± 11 bpm and 163 ± 11 bpm after 4G, 5.5G and 7G exposure, respectively. However, this must be taken with caution due to the impact of high G-exposure on the signal quality for these specific windows. Peak HR was proposed as a possible predictor of G-tolerance during training, as it was dependent on the age, the flying experience and the training status of the military pilot trainees (Tu et al., 2020; Kumar, 2023). The actual changes in SDNN during high G-exposure could not be determined due to the short nature of the measurement and require further investigation and an adapted algorithm for peak identification. Overall results were found to be in the range reported in a recent study, which concluded that the significant change in SDNN during a 5.5G exposure shows that this parameter may be used as a stress predictor and indicator for G-tolerance (Bacevic et al., 2024). However, further comparative validation of the HRV measurements under operationally relevant extreme environments are necessary before investigating associations to contextual performance outcomes such as cognition.

The main goal of this study was to analyze the feasibility of using a textile-based sensor system with a focus on the physiological signals for the monitoring of military pilots during different exposure modalities, such as hypobaric hypoxia, high-G exposure qualification trainings. The sensors were integrated into a textile belt that was worn around the chest. The ECG signals showed very good signal quality: over 90% of the ECG time segments were suitable for further analysis during hypoxia and high-G-force training (**Supplementary Figures 2**, **4**), making them suitable for very accurate analysis of the RR intervals. As only SDNN was evaluated in the present study, our findings cannot be generalized to other HRV metrics. Given that different HRV parameters are likely to be differentially sensitive to signal quality and motion-related artifacts, future studies should systematically investigate the impact of extreme environmental conditions on a broader range of time- and frequency-domain HRV metrics. Recording combined physiological data, such as ECG and respiratory signals, enables a more holistic interpretation of body responses to extreme and even hazardous environmental conditions. The effect of hypoxia was clearly shown by immediate changes in ultra-short heart rate variability and following changes in heart rate, while the respiratory patterns were less conclusive. During high-G-force exposure, respiratory patterns, such as decreased frequency and amplitude, in combination with changes in heart rate, provide objective information about the physical stress the pilot aspirant was exposed to. Overall, some differences in inter-individual signal quality were observed and could come from factors such as individual movement or electrode contact that should be further assessed in future studies. Although the system gained wide acceptance and no discomfort was stated by the participants, to evaluate a holistic feasibility, user feedback and seamless integration into a broader training and in-flight context must be recorded in a quantifiable manner in future studies.

The applicability of such textile-based sensors for continuous monitoring of vital signs of military pilot trainees during training was demonstrated. Such sensors can be potentially directly integrated into the pilot’s flight suit base layers. Further studies are planned to include additional vital signs, such as oxygen saturation and body temperature. In addition, further investigation of chest excursion-based breathing signals is required, especially during hypobaric hypoxia exposure, to improve overall quality and associated thresholds. The data presented here will be complemented by data from additional studies to strengthen these findings. Overall, this work demonstrates the potential of detailed vital sign monitoring in pilots and the development of an early-prediction algorithm for physical and cognitive impairments during training and on-duty flights to increase overall safety and performance.

## Data Availability

The datasets presented in this article are not readily available because they contain sensitive human-subject data. Anonymized data may be shared upon reasonable request after a brief description of the intended use and confirmation of compliance with ethical and legal requirements. Requests can be sent to the corresponding author.
