# Supplementary material for: Monitoring military pilots with textile sensors: physiological responses and signal quality under extreme conditions
Source: Front Physiol. 2026 Sep 14;17:1911780. doi: 10.3389/fphys.2026.1911780 (PMC13617644; doi:10.3389/fphys.2026.1911780)
Supplement: Supplementary file 1 [file DataSheet1.pdf]

Supplementary Material

1 Supplementary Tables

ECG SQ	Mean	SD
Class 1	73.4%	36.2%
Class 2	24%	33.7%
Class 3	0%	0%
Class 4	2.6%	13.8%

Table 1: ECG signal quality classification for hypoxia per interval for n=8 with suitable quality for HRV analysis.

ECG SQ	Mean	SD
Class 1	16.1%	29.8%
Class 2	72.8%	33.7%
Class 3	5.7%	14.8%
Class 4	5.4%	11.5%

Table 2: ECG signal quality classification for high G-Forces per interval for n=8 with suitable quality for HRV analysis.

2 Supplementary Figures

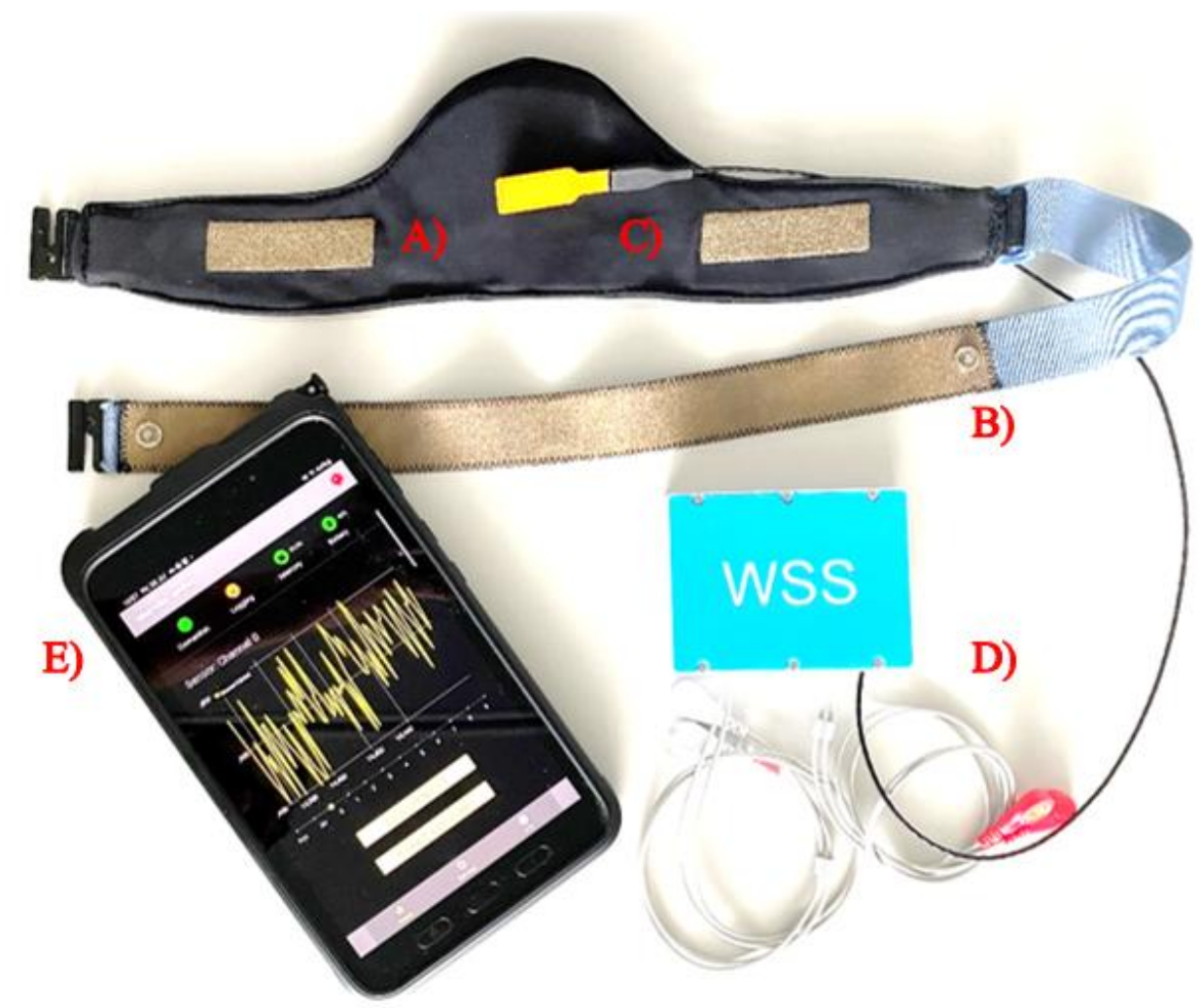

Supplementary Figure 1. Textile monitoring system consisting of knitted electrodes (a) for ECG signal acquisition, a conductive stretch-sensitive textile (b) for the measurement of chest excursions, and a skin heat flux sensor (c; data not considered for further analysis in this study). The textile monitoring system was connected to a custom-made data logger (d) transferring data to a tablet (e) via Bluetooth.

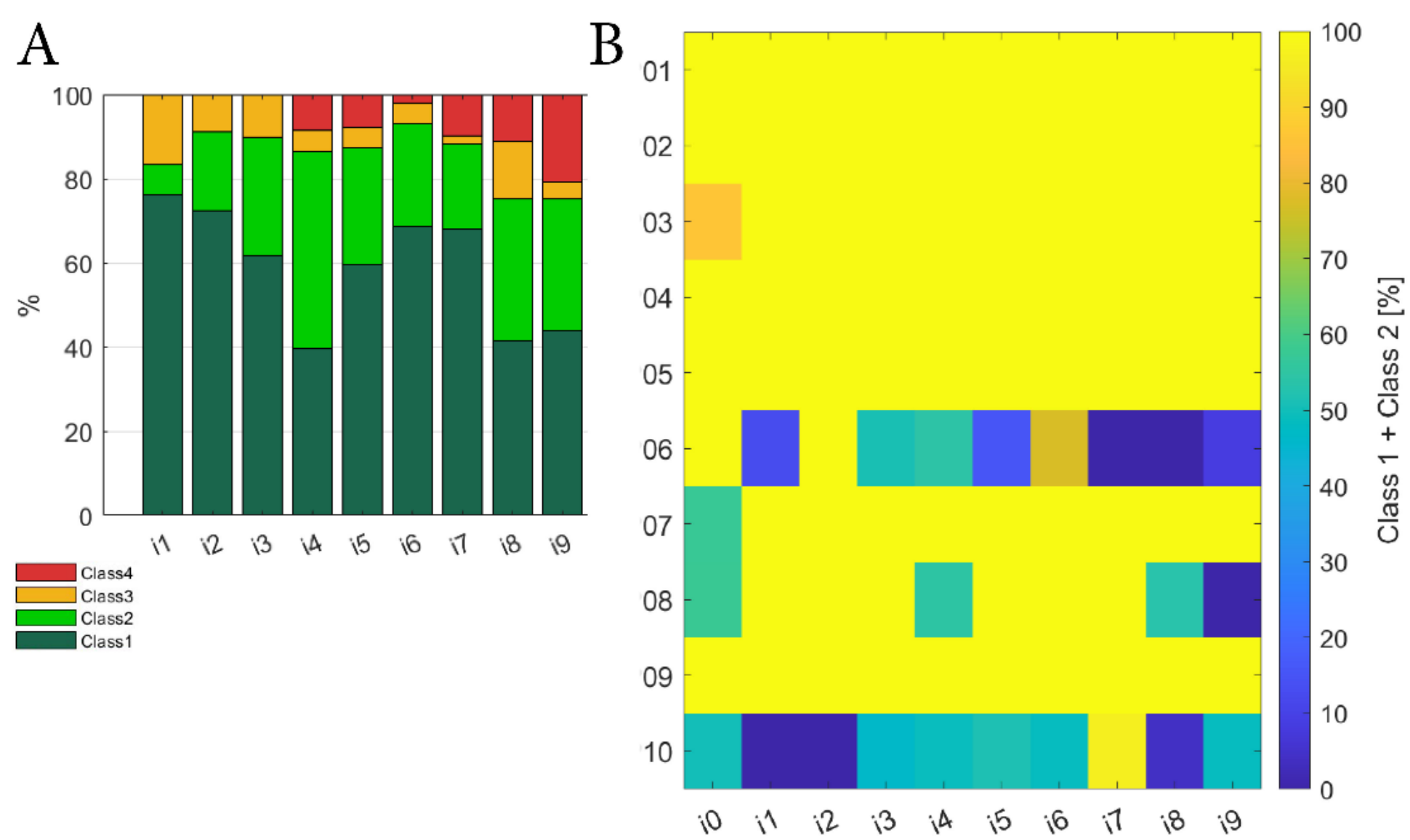

Supplementary Figure 2. SQA ECG Measures during High Altitude Exposure – Hypoxia. A) Classification of ECG signal quality per interval across all subjects. B) Per subject and interval signal quality classification for class 1 and 2 in percentage.

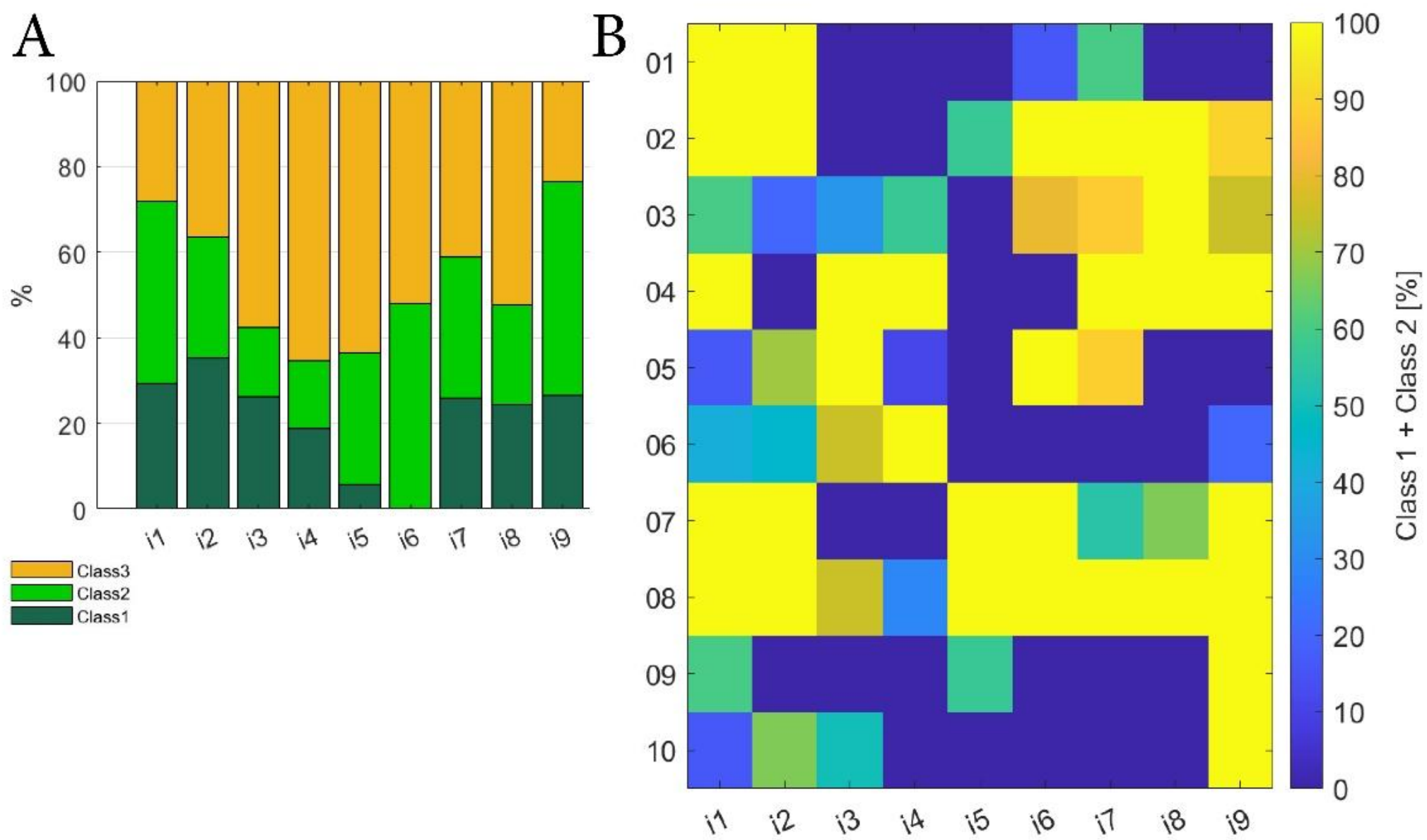

Supplementary Figure 3. SQA BR Measures during High Altitude Exposure – Hypoxia. A) Classification of ECG signal quality per interval across all subjects. B) Per subject and interval signal quality classification for class 1 and 2 in percentage.

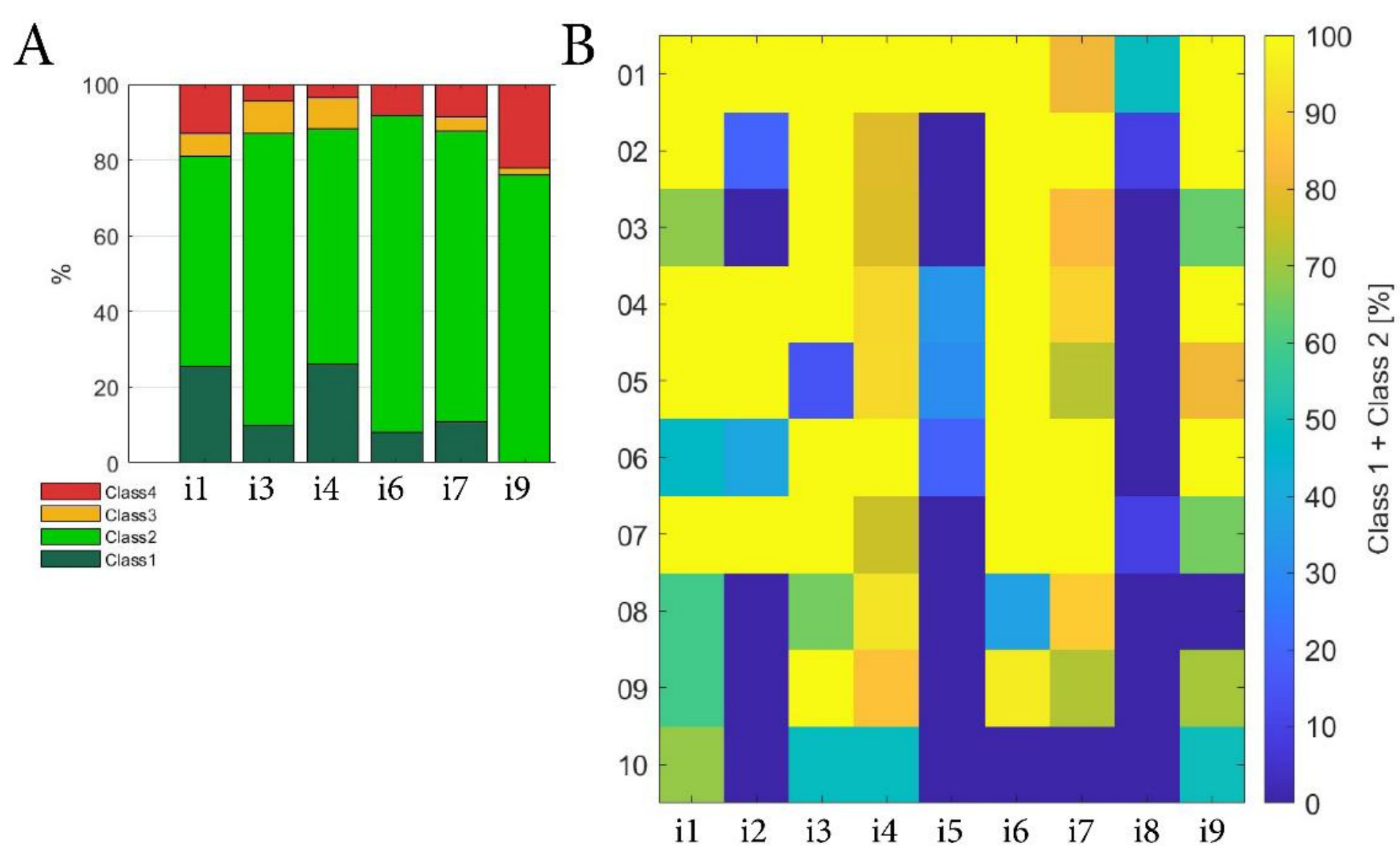

Supplementary Figure 4. SQA ECG Measures during High G – Exposure. A) Classification of ECG signal quality per interval across all subjects. B) Per subject and interval signal quality classification for class 1 and 2.

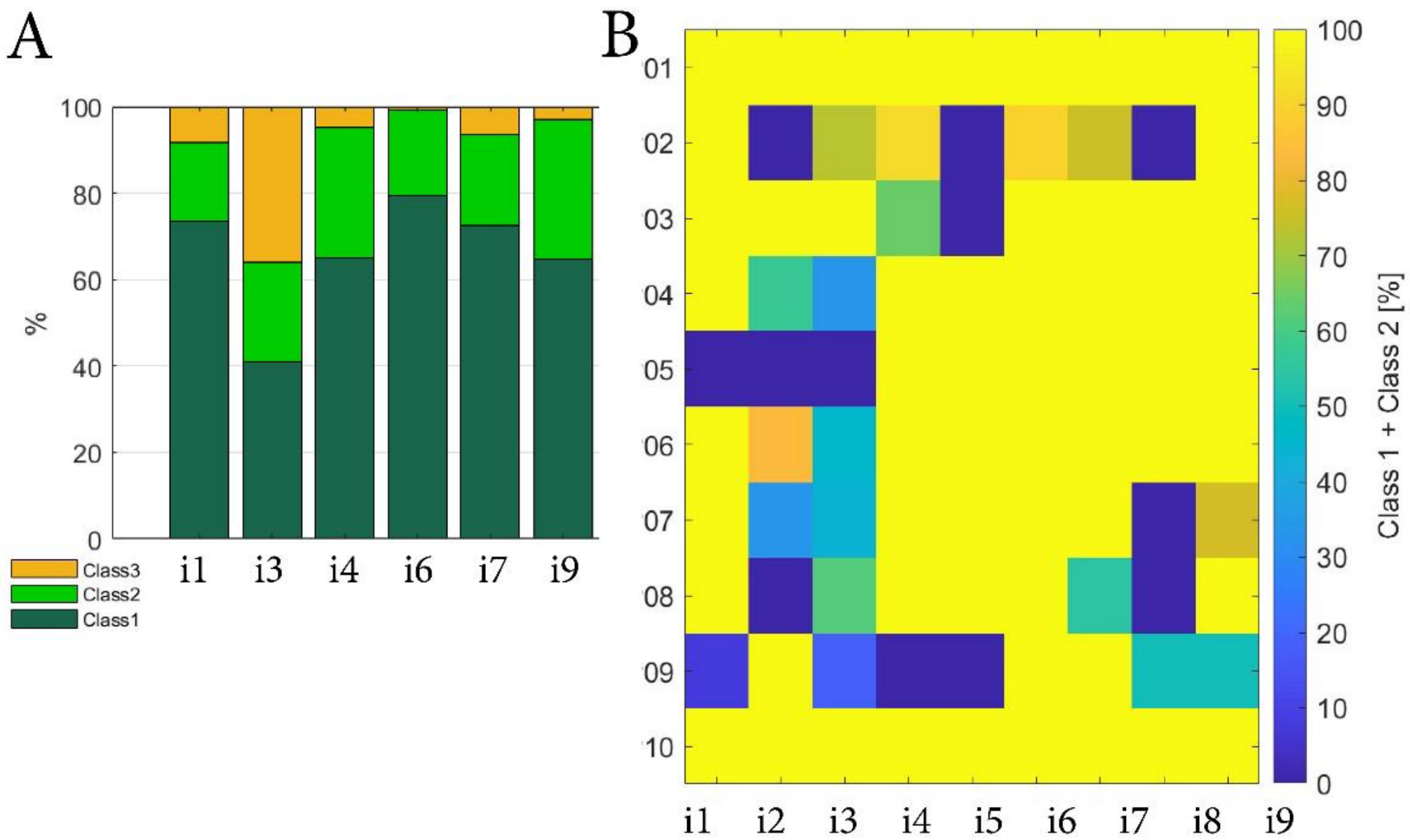

Supplementary Figure 5. SQA BR Measures during High G – Exposure A) Classification of respiratory signal quality per interval across all subjects. B) Per subject and interval signal quality classification for class 1 and 2 in percentage.
